# Adenylyl cyclase 2 expression and function in neurological diseases

**DOI:** 10.1111/cns.14880

**Published:** 2024-07-28

**Authors:** Marsilla Gray, Kevin R. Nash, Yao Yao

**Affiliations:** ^1^ Department of Molecular Pharmacology and Physiology, Morsani College of Medicine University of South Florida Tampa Florida USA

**Keywords:** Adcy2, cAMP, neurodegenerative disease, psychiatric disorder, stroke

## Abstract

Adenylyl cyclases (Adcys) catalyze the formation of cAMP, a secondary messenger essential for cell survival and neurotransmission pathways in the CNS. Adcy2, one of ten Adcy isoforms, is highly expressed in the CNS. Abnormal Adcy2 expression and mutations have been reported in various neurological disorders in both rodents and humans. However, due to the lack of genetic tools, loss‐of‐function studies of Adcy2 are scarce. In this review, we summarize recent findings on Adcy2 expression and function in neurological diseases. Specifically, we first introduce the biochemistry, structure, and function of Adcy2 briefly. Next, the expression and association of Adcy2 in human patients and rodent models of neurodegenerative diseases (Alzheimer's disease and Parkinson's disease), psychiatric disorders (Tourette syndrome, schizophrenia, and bipolar disorder), and other neurological conditions (stress‐associated disorders, stroke, epilepsy, and Lesch‐Nyhan Syndrome) are elaborated. Furthermore, we discuss the pros and cons of current studies as well as key questions that need to be answered in the future. We hope to provide a focused review on Adcy2 that promotes future research in the field.

## Graphical abstract

Mutations and expression changes of adenylyl cyclase 2 (Adcy2), a CNS‐enriched enzyme that converts ATP to cAMP, are associated with several neurodegenerative and psychiatric disorders.

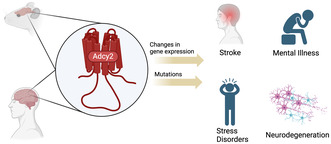

## 1 INTRODUCTION

**FIGURE 1:**
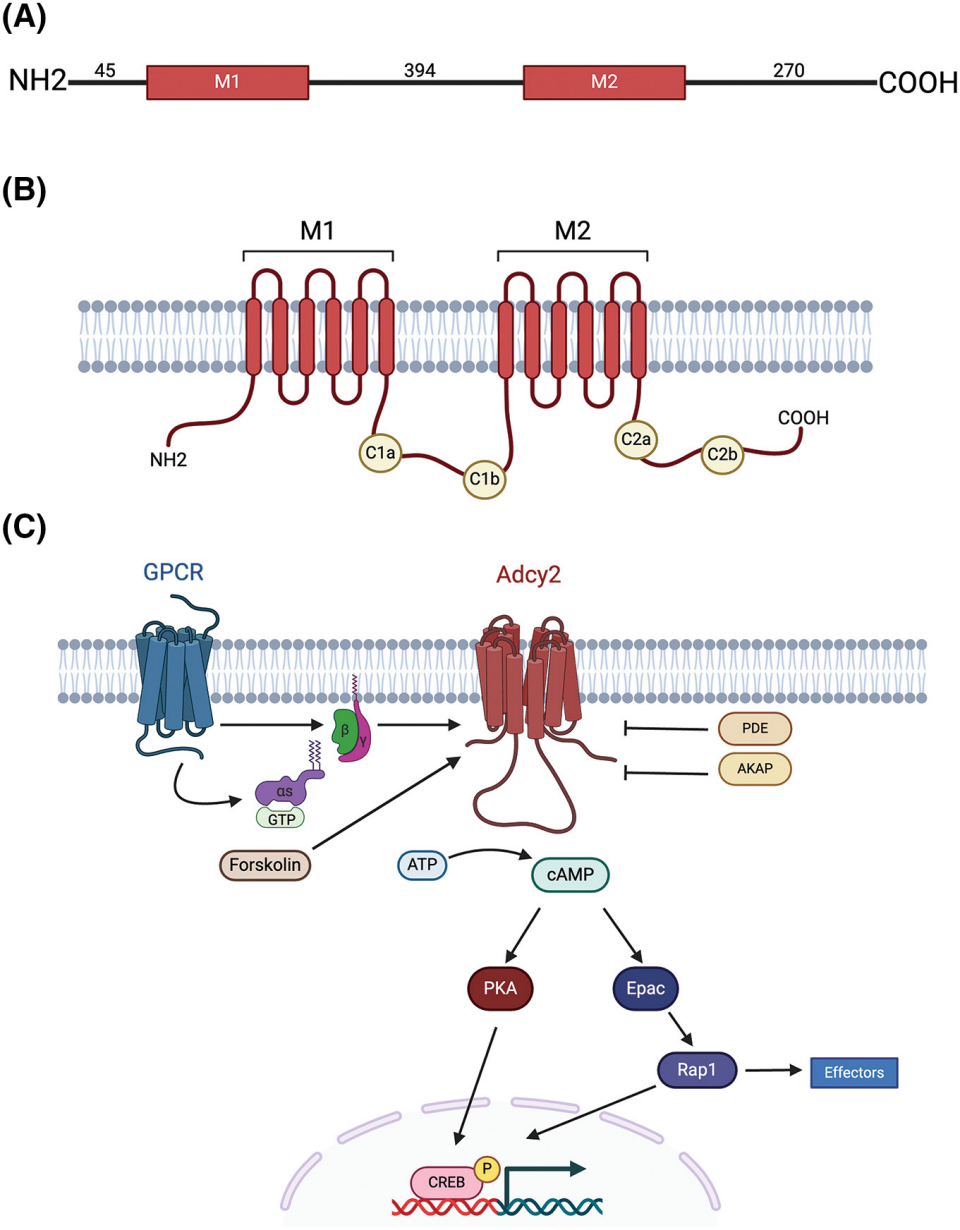
Adenylyl cyclase 2 (Adcy2) structure and classical signaling pathways. (A) Linearized Adcy2 structure with M1/M2 transmembrane domains highlighted. (B) Intermembrane Adcy2 structure with M1/M2 transmembrane domains (red), C1a/C2a catalytic sites (yellow), and C1b/C2b regulatory and dimerization domains (yellow). (C) Classical Adcy2 signaling pathways. G protein as well as PDEs and AKAP regulate the production of cAMP, which controls transcription via PKA and/or Epac/Rap1 activity. Rap1 can also interact with downstream effectors directly. Created with BioRender.

Adenylyl cyclases (Adcys) are a family of enzymes that catalyze the formation of cyclic adenosine 3′,5′‐monophosphate (cAMP) from ATP.^1^ cAMP is a vital second messenger that can act either in a kinase‐dependent manner via protein kinase A (PKA) or in a kinase‐independent manner via exchange protein directly activated by cAMP (Epac).^2^ Each Adcy consists of two transmembrane helical regions (M_1_ and M_2_) and two cytoplasmic regions (C_1_ and C_2_), which are further divided into C_1a_, C_1b_, C_2a_, and C_2b_ (Figure 1). These cytoplasmic regions contain the catalytic and dimerization domains.^3^

Mammalian Adcys are grouped into five families based on their structure, function, and regulatory properties (Table 1).^1^ Most can be regulated by G‐protein‐coupled receptors (GPCRs) via interactions with their stimulatory (G_αs_) and inhibitory (G_αi/o_) G_α_ and/or dimerized G_βɣ_ subunits.^4^ Group I Adcys (Adcy1, 3, and 8) are stimulated by Ca^2+^/calmodulin (CaM) and G_αs_, but inhibited by G_αi/o_ and G_βɣ_. Group II Adcys (Adcy2, 4, and 7) are stimulated by G_αs_ and G_βɣ_, but insensitive to Ca^2+^/CaM. Group III Adcys (Adcy5 and 6) are stimulated by G_αs_ and G_βɣ_ but inhibited by G_αi/o_ and Ca^2+^. Group IV has only one member (Adcy9) and is activated by G_αs_, inhibited by calcineurin and PKC, and insensitive to forskolin.^1^, ^5^ The last group consists of Adcy10, also known as soluble Adcy (sAdcy), a unique soluble isoform that possesses the conserved catalytic domain. Unlike other groups, sAdcy cannot be activated by G proteins, is insensitive to forskolin, but can be activated by Ca^2+^ and bicarbonates.^1^, ^5^, ^6^

**TABLE 1:** Summary of Adcy isoforms and effectors.

Family	Protein	Effectors		
		Stimulating	Inhibiting	Insensitive
Group I	Adcy1	Gαs, Ca2+/CaM, forskolin	Gai/o, Gβɣ	
	Adcy3			
	Adcy8			
Group II	Adcy2	Gαs, Gβɣ, forskolin		Ca2+/CaM
	Adcy4			
	Adcy7			
Group III	Adcy5	Gαs, Gβɣ, forskolin	Gai/o, Ca2+/CaM	
	Adcy6			
Group IV	Adcy9	Gαs	Calcineurin, PKC	Forskolin
Group V	Adcy10	Ca2+/CaM, bicarbonates		Forskolin

*Note*: All Adcys are membrane‐bound except Adcy10/sAdcy.

All Adcys, except Adcy4, have been associated with central nervous system (CNS) disorders including anxiety, schizophrenia, bipolar disorder, depression, post‐traumatic stress disorder, and autism in both rodent and human studies.^1^, ^7^, ^8^, ^9^, ^10^, ^11^, ^12^, ^13^

Among all Adcys, Adcy2 is relatively less studied. Adcy2 is abundantly and widely expressed in the CNS, and several studies have found an association of Adcy2 with multiple neurodegenerative and psychiatric conditions.^1^, ^5^, ^14^, ^15^, ^16^ In this review, we focus on the functions of Adcy2 in various neurological diseases, with the hope of stimulating interest and promoting further research in this field. First, we briefly introduce the structure, expression, and function of Adcy2 in the CNS. Next, we describe recent findings on the roles of Adcy2 in neurodegenerative disorders, psychiatric diseases, and other neurological conditions. Finally, we discuss the pros and cons of previous studies and elucidate key questions that need to be answered in the future.

## 2 ADCY2 STRUCTURE, EXPRESSION, AND FUNCTION

The human *Adcy2* gene contains 26 exons and is mapped to a chromosomal region of observed homology between mouse and humans.^17^ It has no TATA‐box but contains a putative GC box that can be regulated by transcription factor specificity protein 1 (Sp1).^18^ The *Adcy2* gene encodes a 1090‐amino acid protein with a molecular weight of approximately 123 kDa.

Rodent and human studies find that *Adcy2* mRNA is primarily expressed in the brain.^19^, ^20^, ^21^ In the developing brain, *Adcy2* is found in the mamillary body of the hypothalamus and in scattered cholinergic cells of the striatum.^19^, ^22^, ^23^ In adult brains, *Adcy2* is detected in all layers of the olfactory bulb, layers 2–4 of the cortex, and CA1 region of the hippocampus.^22^ In addition, in‐situ hybridization analysis reveals strong expression of *Adcy2* in CA1 and CA4 of the hippocampus, granule cells of the cerebellum, superior and inferior colliculi, and the brain stem.^24^ Cell‐specific expression studies show that *Adcy2* is expressed at high levels in astrocytes, arterial smooth muscle, and oligodendrocyte precursor cells, while at lower levels in neurons, endothelial cells, and microglia.^25^, ^26^, ^27^, ^28^

Adcy2 catalyzes the production of cAMP, which acts indiscriminately on PKA and Epac to influence a multitude of cellular processes including adhesion, movement, and differentiation.^2^ Within the CNS, PKA and Epac are involved in the regulation of various functions including microglial cytokine production, learning and memory, axon growth, and myelination.^29^, ^30^, ^31^, ^32^, ^33^ Adcy2 has also been reported as an NAD‐binding protein, a family of proteins associated with multiple neurodegenerative disease‐related pathways.^34^

As a member of the group II Adcys, Adcy2 is insensitive to Ca^2+^/CaM, but can be stimulated by G_αs_ & G_βɣ_. It has been shown that Adcy2 can be regulated by phosphodiesterases (PDEs) and A‐kinase anchoring proteins (AKAPs).^35^ For example, AKAP9, also known as Yotiao, directly inhibits Adcy2 via the N‐terminus, increasing cAMP signal specificity.^36^

## 3 ADCY2 IN NEURODEGENERATIVE DISORDERS

### 3.1 Alzheimer's disease

Alzheimer's disease (AD) is the largest cause of dementia in the U.S., affecting approximately 6.7 million individuals over 65 years of age in 2023.^37^ AD is characterized by the presence of extracellular amyloid‐β (Aβ) plaques and intracellular tau tangles spreading from the hippocampus to the cortex in a slow propagative manner.^38^, ^39^ Aβ/tau accumulation is accompanied by neurodegeneration, abnormal cerebrovascular morphology, blood‐brain barrier (BBB) dysfunction, glial activation, and mitochondrial stress.^39^, ^40^, ^41^

Studies have linked Adcy2 to AD and AD comorbidities. However, how exactly Adcy2 levels change in AD brains is controversial. On one hand, a negative correlation of Adcy2 with AD has been found in OXYS rats, an inbred strain with an accelerated aging phenotype that spontaneously develops AD‐like pathology, including cognitive deficits, hippocampal neuronal degeneration, oxidative and mitochondrial stress, and Aβ accumulation.^42^ Specifically, the number of hippocampal neurons decreases rapidly with age in OXYS rats, and this hippocampal neurodegeneration coincides with the downregulation of *Adcy2*.^42^ It should be noted that *Adcy2* mRNA levels are downregulated in both Wistar control and OXYS brains at 5–18 months compared to the juvenile age.^43^ It remains unclear whether Adcy2 expression is further reduced in OXYS brains at 5–18 months compared to age‐matched controls. Thus, it is possible that reduced Adcy2 expression is a consequence of aging rather than AD pathology. Direct evidence linking Adcy2 reduction to AD pathogenesis is lacking. Currently, it is unknown how Adcy2 level alters in other mouse models of AD, such as the 5xFAD model of Aβ pathology and PS19 model of tauopathy.

On the other hand, there are also studies showing a positive correlation between Adcy2 and AD. For example, a microarray dataset reports that *Adcy2* is upregulated in the hippocampus of patients with severe AD (mean Braak stage 5.9).^44^, ^45^ This direct evidence highlights a possible role of Adcy2 in AD pathogenesis. Given the role of cAMP in chemoattractant signaling and leukocyte extravasation in the CNS, increased *Adcy2* in the hippocampus may indicate neurons in distress signaling for immune support.^46^, ^47^

*Adcy2* has been identified as a risk gene with a high number of single‐nucleotide polymorphisms (SNPs) between AD and cognitive normal controls.^14^, ^48^ These SNPs may affect Adcy2 functions, including vascular smooth muscle contraction, gap junction function, purine metabolism, chemokine signaling, and calcium signaling pathways, all of which become dysfunctional in AD.^49^ The functional significance of these SNPs in AD pathogenesis needs future studies. Besides SNPs, decreased *Adcy2* methylation is found in AD and mild cognitive impairment (MCI) patients compared to healthy controls, with the greatest effects observed in males.^50^ Decreased methylation of *Adcy2* may contribute to the previously discussed upregulation of hippocampal *Adcy2* in AD patients. It should be noted, however, that despite the upregulation of hippocampal Adcy2, cAMP response element binding protein (CREB), the transcription factor that regulates genes involved in learning and memory, is impaired in AD patients.^51^ What causes the increased expression of Adcy2 is currently unknown.

Although there is no direct evidence linking Adcy2 to Aβ accumulation, Adcy2 is associated with amyloid precursor protein (APP) under acute stress conditions. It has been shown that inhibiting *Adcy2* expression following acute stress reduces APP levels in DBA/2 J mice,^52^ an inbred strain often used in aging and sensorineural studies. Subsequent studies show that cAMP derived from forskolin‐sensitive Adcys, including Adcy2, stimulates APP production in neurons and astrocytes via adrenergic receptor activation.^53^, ^54^ As a risk factor for dementia, chronic stress may increase Adcy2 expression and cAMP signaling, leading to elevated APP levels and consequently Aβ accumulation.^55^ However, a direct association between Adcy2 and stress preceding dementia has not been established.

Cerebral amyloid angiopathy (CAA), a risk factor for dementia and comorbidity of AD, is characterized by deposition of Aβ into cerebral blood vessels and decreased vessel integrity.^56^ Enrichment of *Adcy2* is also observed in humans with spontaneous CAA.^57^ This indirect evidence suggests that Adcy2 may be involved in cerebrovascular pathology in AD brains. Echoing this finding, cAMP is increased in cerebral microvessels of AD patients compared to non‐demented elderly controls, whereas comparable cAMP levels are observed in young and old wildtype rats.^58^ These results suggest that cAMP upregulation is caused by AD pathology rather than normal aging.

### 3.2 Parkinson's disease

Parkinson's disease (PD), first described in 1817, is a neurodegenerative disorder characterized by motor defects such as tremor and rigidity.^59^ Key pathological changes of PD include loss of dopaminergic neurons in the substantia nigra, α‐synuclein aggregation, mitochondrial dysfunction, neuroinflammation, and impaired protein clearance.^59^

Although the functional significance of Adcy2 in PD pathogenesis remains largely unknown, a negative correlation between Adcy2 and PD has been reported. First, Adcy2 is downregulated in the striatum of mice treated with 1‐methyl‐4‐phenyl‐1,2,3,6‐tetrahydropyridine (MPTP), a widely used mouse model of PD.^60^ It should be noted that the downregulation of Adcy2 in PD brains may be simply due to the loss of dopaminergic neurons, since dopamine can activate cAMP signaling through dopamine receptors.^61^ Next, in humans, *Adcy2* has been identified as a gene of interest in several key signaling pathways whose dysfunction increases PD risk, such as calcium signaling and glutaminergic synapse.^62^, ^63^ In addition, PANTHER pathway analysis indicates that downregulation of *Adcy2* and other genes may be responsible for the altered serotonin degradation and dopamine signaling in the subventricular zone of PD patients.^64^

In a recent GWAS, an SNP in *Adcy2* was identified as a genetic factor that makes PD patients susceptible to levodopa‐induced dyskinesia (LID).^65^ LID presents as abnormal movements such as stereotypic, choreiform, and throwing movements as well as dystonia that mainly involves the head, face, limbs, and trunk. It has been found that neuronal knockdown of *Adcy2* resolves LID in a Drosophila model of PD by suppressing dopamine‐like receptor 1 (D1).^65^ However, in a rat model of PD with chronic levodopa administration, *Adcy2* is downregulated compared to acute and untreated controls.^66^ In the rat model, the reduction in *Adcy2* may represent a compensation mechanism. This aligns with a theory that precise regulation of cAMP turnover in the striatum is necessary to prevent abnormal movement.^65^, ^67^ Consistent with this, both gain‐ and loss‐of‐function mutations in Adcys cause imbalances in cellular cAMP levels and contribute to movement disorders.^65^, ^68^ The previously mentioned SNP in *Adcy2* may enhance its activity and cause overactive cAMP signaling in motor pathways, similar to mutations in the cytoplasmic region of Adcy5 in familial dyskinesia, which include gain‐ and loss‐of‐function mutations with regional severity.^68^ Mutations in the regulatory domains result in less severe symptoms and those in the catalytic domains more severe.^68^ Increased D1 signaling as a result of levodopa administration causes increased Adcy activity, and in the case of Adcy2, this may result in overstimulation of the enzyme with already enhanced function.

## 4 ADCY2 IN PSYCHIATRIC DISEASES

### 4.1 Tourette syndrome

Tourette syndrome (TS) is a chronic neurodevelopmental disorder characterized by uncontrollable motor and vocal tics. TS has a childhood onset between 4 and 8 years of age, and tics generally improve or resolve in adulthood.^69^, ^70^ Little is known about the pathophysiology of TS, but differences in axonal pruning during development and dysfunction in the reward and sensorimotor circuits of the striatum are thought to contribute.^69^ TS is also commonly comorbid with other disorders, including obsessive‐compulsive disorder (OCD) and attention‐deficit hyperactivity disorder (ADHD).^69^, ^70^

Current studies suggest that Adcy2 may play a dual role in TS. On one hand, there is evidence supporting a beneficial role of Adcy2. Loss of an intron/exon splice site in *Adcy2* and recurrent deletion of a subunit of cAMP‐activated PKA have been identified in TS.^15^, ^71^ These mutations reduce cAMP supply to neurons, which in turn prevents retrograde mitochondrial transport. In addition, they also decrease PKA signaling, which downregulates mitochondrial fission.^15^ Given the important functions of mitochondrial transport and dynamics in neuronal function,^15^, ^72^ it is reasonable to speculate that restoring Adcy2/cAMP signaling exerts a beneficial role in TS.^71^

There are also studies reporting a detrimental role of Adcy2. While hyperdopaminergic cortico‐striatal activity was previously thought to underlie TS etiology, newer evidence instead suggests that inhibited dopaminergic signaling drives TS pathology.^69^, ^73^, ^74^ Stimulation of D2 receptors, which inhibits Adcy function and downregulates cAMP production,^75^ has been found to relieve tics in both child and adult patients,^73^, ^74^ highlighting a detrimental role of Adcy2/cAMP in TS.

One possible explanation for the distinct roles of Adcy2 in TS is its subcellular location. Mitochondria in neurons are mainly found in the axons, whereas D2 receptors are predominantly found at the synapses. It is likely that Adcy2 may exert a beneficial role in axons, but a detrimental role in synapses. The exact mechanism underlying the dual role of Adcy2 in TS needs further investigation.

### 4.2 Schizophrenia

Schizophrenia (SZ) is a psychiatric disorder with positive symptoms (e.g. delusions and audio‐visual hallucinations), negative symptoms (e.g. lack of emotionality), and cognitive decline. Impairment in social interactions and self‐care often precede the negative or positive symptoms.^76^ Developmental and early childhood conditions, decreased synaptic pruning, and genetic variants are all thought to contribute to SZ.^76^, ^77^

In a rat model of SZ, reduced *Adcy2* expression has been found in various brain regions. Specifically, lower levels of *Adcy2* are detected in the nucleus accumbens (NAc), an important structure that coordinates emotional reaction, and the prefrontal cortex of juvenile SZ rats.^78^ In adult SZ rats, such reduction of *Adcy2* is only observed in the NAc.^78^ Since Adcy2 is a downstream target of dopaminergic and glutamatergic signaling in the NAc, its reduction may contribute to the negative symptoms of SZ by downregulating emotional responsiveness.^79^, ^80^

Recently, an SNP (rs58502974) in *Adcy2* has been identified as a susceptibility factor for SZ in an Iranian population with the AA allele being highly associated with SZ, while the T allele is protective.^16^ In addition, AKAP9, a direct inhibitor of Adcy2 that regulates cAMP signal specificity,^36^ has been linked to SZ in a recent GWAS.^81^ Specifically, the K873R single‐nucleotide variation (SNV) on AKAP9, which has a possibly damaging role, has been identified in 4 out of 572 SZ cases.^81^ It remains unclear how this SNV, which occurs in the Adcy2 binding region, affects the inhibitory effect of AKAP9 on Adcy2 activity.^81^ Since AKAP9 expression is highest in retinal photoreceptor cells,^82^, ^83^ it is speculated that decreased AKAP9 inhibition of cAMP in these cells may cause overactive phototransduction and regulation of light/dark specificity, contributing to the positive symptom of visual hallucinations.^84^

### 4.3 Bipolar disorder

Bipolar disorder (BD) is an inheritable chronic mood disorder with a high risk of suicide. Patients with BD have episodes of major depression or mania and often possess comorbidities of anxiety and substance abuse disorders.^85^, ^86^ The high rate of heritability indicates a prominent genetic component. While multiple genes have been associated with BD via GWAS, none have been determined as causative or to carry major risk for BD thus far.^86^

Knowledge on Adcy2 in BD predominantly comes from human studies. One study shows that Adcy2 is associated with early‐onset BD when dysfunction in serotonin and dopamine signaling arise.^87^ This association is also seen in the same pathways in ADHD.^87^ Another report finds that *Adcy2* is upregulated in both astrocytes and neurons in BD patients.^88^ In addition, a missense variant (rs13166360) in *Adcy2* is associated with general BD risk, but some polymorphisms are associated with severity of the disease.^89^ For example, Iranian BD patients with the C allele of the rs2290910 polymorphism are more likely to suffer suicidal ideation, and those with the T allele are more likely to attempt suicide.^90^ Similarly, several other polymorphisms have been associated with the onset or incidence of BD in male patients in Chinese Han population.^91^ Interestingly, there are no gender differences in the onset, severity, or symptoms of BD,^86^, ^92^ suggesting that while genetics may predispose an individual to BD, other factors also contribute to its pathogenesis.

## 5 ADCY2 IN OTHER NEUROLOGICAL CONDITIONS

### 5.1 Stress‐associated disorders

Multiple neurodegenerative and psychiatric conditions have been associated with stress, which acts as either a major causative or risk factor during disease development.^93^, ^94^ Adcy2 has not been examined within the context of acute or chronic stress models, but differential gene expression has been observed in stress‐associated disorders.

Stress has been identified as a risk factor for dementias, including AD. Human data shows that exposure to stress early in life is correlated with a higher risk of dementia.^55^, ^95^ Although Adcy2 is associated with APP levels under acute stress conditions (see Alzheimer's Disease section), its role in stress remains largely unknown.

Anxiety and depression are common stress‐associated disorders. One study found significantly decreased *Adcy2* in the hippocampus and prefrontal cortex in young males of a mouse model of anxiety.^96^ Echoing this finding, ego network analysis identified *Adcy2* as a top candidate gene related to anxiety disorders with the catechol‐O‐methyltransferase (*Comt1*) overexpression phenotype.^97^ Interestingly, *Adcy2* is similarly dysregulated, among other retrograde endocannabinoid signaling pathway genes, in patients with major depressive disorder (MDD), although the directionality of gene expression is not stated in this study.^98^

Substance abuse disorders, including alcohol use disorder (AUD), are frequently comorbid with anxiety and stress disorders.^99^, ^100^ It has been reported that *Adcy2* is downregulated in the hippocampus of rats with an innate preference for alcohol.^101^ There are currently no data on a specific role of Adcy2 in AUD. However, given the important role of Adcy2 in regulating neurotransmitters in the NAc, a region heavily involved in the reward system and substance abuse disorders, it is reasonable to hypothesize that Adcy2 participates in the pathogenesis of AUD.

### 5.2 Stroke

Stroke is one of the leading causes of death and disability worldwide. It is caused by either a lack of blood supply to the brain (ischemic stroke) or bleeding into the brain (hemorrhagic stroke).^102^, ^103^ Although subtype‐specific features exist, both ischemic stroke and hemorrhagic stroke share common pathologies including excitotoxicity, neuroinflammation, oxidative stress, and angiopathy.^103^

Current studies on Adcy2 in stroke mainly come from ischemic stroke. In a GWAS, a gene locus associated with factor VII‐activating protease (FSAP) activity (rs35510613) was identified 19 kb upstream of *Adcy2* in ischemic stroke patients.^104^, ^105^ Two SNPs (rs12652415 and rs1609428) located in an intron of *Adcy2* in Swedish populations are thought to interact with rs35510613 to regulate FSAP activity.^105^ Interestingly, an in vitro study suggests that increased cAMP levels upregulate transcription of *Habp2*, the gene encoding FSAP, via PKA activation.^105^ Thus, *Adcy2* may upregulate FSAP expression, and individuals with the rs12652415 and rs1609428 SNPs in *Adcy2* may further enhance FSAP enzymatic activity. Subsequent loss‐of‐function studies show that genetic ablation of FSAP increases neointima and leukocyte recruitment following vascular injury in skeletal muscle in mice, whereas inhibition of FSAP improves BBB integrity and ischemic stroke outcomes in humans.^104^, ^106^ Whether this difference is species (rodent vs. human)‐ and/or tissue (muscle vs. brain)‐dependent is currently unknown and should be determined in future research.

Currently, there are no studies on the expression and function of Adcy2 in hemorrhagic stroke.

### 5.3 Epilepsy

Epilepsy is a disease where patients have multiple seizures within 24 hours, or the 10‐year risk of seizure is >60% in affected individuals.^107^ *Adcy2* mRNA is downregulated in the hippocampus of both individuals with temporal lobe epilepsy and mouse models of status epilepticus.^108^ Interestingly, a proteomics study reports upregulated hippocampal Cox2, an enzyme that stimulates Adcy2 activity, in a rat model of temporal lobe epilepsy.^109^ Consistent with this observation, cAMP is proconvulsive when injected into rodents' brains,^110^, ^111^ and a previous study found that a region of mouse chromosome 13 including *Adcy2* contains a candidate gene(s) for audiogenic seizure development.^112^ The discrepancy between mRNA and protein expression in human data and model species may be caused by different detection approaches (mRNA vs. proteins). Therefore, it is essential to investigate Adcy2 changes at both mRNA and protein levels in future research.

### 5.4 Lesch‐Nyhan syndrome

Lesch‐Nyhan syndrome (LNS) is an X‐linked developmental disorder characterized by dysfunctional purine metabolism, mental retardation, and uncontrollable self‐injury.^113^ In the hypoxanthine phosphoribosyl transferase (HPRT)‐deficient rat B103 neuroblastoma model of LNS, Adcy2 is almost completely absent,^114^ indicating a possibly important role of Adcy2 in LNS. However, the relationship between Adcy2 and LNS needs to be validated in more sophisticated models (e.g. in vivo) in the future.

## 6 CONCLUSION AND FUTURE DIRECTIONS

Adcy2, converting ATP to second messenger cAMP in cells, regulates multiple important functions. In the CNS, dysfunction of Adcy2 is observed in multiple neurological diseases, including neurodegenerative disorders, psychiatric diseases, and other neurological conditions. It seems that changes in Adcy2 expression levels are predominantly found in neurodegenerative disorders, whereas Adcy2 SNPs are more common in psychiatric diseases. Regardless of Adcy2 variant, it often coincides with dysfunctional dopaminergic, GABAergic, and serotonergic signaling, since it is a downstream target of postsynaptic GPCRs in these signaling pathways. Adcy2 mutations and expression changes in various neurological diseases are summarized in Table 2.

**TABLE 2:** Adcy2 mutations and dysregulation in neurological diseases.

Diseases	Changes/mutations	Types	Regions	Species	Ages	References
AD	Downregulation	RNA	HPC	Rat	5–18 mo	42, 43
	Upregulation	RNA	HPC	Human	80+ yo	44, 50
	Various SNPs	RNA		Human		14, 48
	Upregulation (CAA)	RNA		Human		^57^
PD	Downregulation	RNA	SVZ	Human	76 yo^a^	^64^
		Protein	Striatum	Mouse	2 mo	^60^
TS	Loss of intron/exon splice site	RNA		Human		^15^
SZ	Downregulation	RNA	NAc, PFC	Rat	45 days	^78^
		RNA	NAc	Rat	90 days	^78^
	rs58502974	RNA		Human		^16^
BD	Upregulation	RNA	Astrocytes, neurons	Human	55 yo^a^	^88^
	rs58502974	RNA	Intronic	Human		^87^
	rs13166360	RNA	Intronic	Human		^89^
	rs2290910	RNA		Human		^90^
	rs2892609, rs4702484, rs1460970, rs1032717, rs1027579, rs1027579, rs10059539, rs10462841, rs326149, rs326174, rs1560172, rs6864771, rs1864071, rs326175	RNA		Human^b^		^91^
Stress‐associated disorders	Downregulation (Anxiety)	RNA	HPC, PFC	Mouse^b^	15–17 wo	^96^
	Downregulation (AUD)	RNA	HPC	Rat^b^	90‐100 days	^101^
Ischemic stroke	rs12652415, rs1609428	RNA	Intronic	Human	58 yo^c^	^105^
Epilepsy	Downregulation	RNA	HPC	Human		^108^
	Downregulation	RNA	HPC	Mouse	8–12 wo	^108^
LNS	Downregulation	RNA		Rat		^114^

Abbreviations: AD, Alzheimer's disease; AUD, alcohol use disorder; BD, bipolar disorder; CAA, cerebral amyloid angiopathy; HPC, hippocampus; LNS, Lesch‐Nyhan syndrome; NAc, nucleus accumbens; PD, Parkinson's disease; PFC, prefrontal cortex; SVZ, subventricular zone; SZ, schizophrenia; TS, Tourette syndrome.

^a^ Mean age of subjects.

^b^ Males.

^c^ Median age of subjects.

Although significant progress has been made in identifying Adcy2's involvement in neurological diseases, several key questions remain unanswered. First, it remains unclear whether *Adcy2* alteration or its SNPs identified in neurological disorders is causative or secondary to other changes. Temporal characterization of Adcy2 levels together with loss/gain‐of‐function studies will provide insights on this important question. Although there are some reports in these aspects, this information is largely unknown and needs future research. Second, what is the function of Adcy2 in the pathogenesis of each neurological disorder? This question is largely unanswered partially due to a lack of genetic models for *Adcy2* research. Many previous studies used indirect readout of Adcy2 activation, such as G_αs_/G_βɣ_ dependence, which is not accurate since it can apply to other group II Adcys.^115^ Although Adcy2 global knockout mice have been generated and are being investigated currently, tissue‐specific knockouts and inducible knockouts are not available. In addition, Adcy2 reporter mice and tissue‐specific/inducible Adcy2 overexpression mouse lines are not available either. The lack of these genetic tools makes cell‐specific and/or controllable manipulation of Adcy2 expression in vivo impossible, which presents significant challenges in the field. Developing these genetic tools will enable in‐depth functional studies and shed light on the significance of Adcy2 in neurological disorders. Third, is there functional compensation between Adcy2 and other Adcys? Given the existence of multiple Adcy isoforms, it is reasonable to believe there is functional redundancy. However, the specific Adcy isoforms involved in compensation remain largely unknown, which relies on our understanding of Adcy expression in each cell type under both physiological and pathological conditions. Compound knockout mice, in which multiple Adcy isoforms are ablated, may be useful in determining functional redundancy in vivo. Fourth, is the function of Adcy2 completely dependent on its adenylyl cyclase activity? Although Adcy2 acts on ATP and converts it to cAMP, we cannot exclude the possibility that it has adenylyl cyclase‐independent functions. Adcy2 mutants lacking adenylyl cyclase activity may be used to answer this question. Last, it is essential to validate results from animal studies in human samples. There are multiple documented differences in *Adcy2* expression between rodent models of disease and human patients. One possible reason is different methods and/or brain regions used in these studies. It should be noted that mRNA changes do not always echo alterations at the protein levels. Another possible reason is species difference. Rodents can replicate some, but not all, disease pathology in human patients. Thus, key findings from animal research should always be validated in human cells or tissues.

Answers to these questions will enable a comprehensive understanding of the expression and function of Adcy2 in neurological diseases, which will substantially move the field forward.

## AUTHOR CONTRIBUTIONS

MG drafted the manuscript. KRN and YY commented and revised the manuscript. All authors read and approved the manuscript.

## FUNDING INFORMATION

This work was partially supported by National Institutes of Health grants (R01HL146574, RF1AG065345, R01NS134134, R21AG073862, and R21AG064422) to YY. This publication was made possible by an NHLBI‐funded predoctoral fellowship to MG (T32HL160529).

## CONFLICT OF INTEREST STATEMENT

The authors declared no potential conflicts of interest with respect to the research, authorship, and/or publication of this article.

## Data Availability

Data sharing is not applicable to this article as no new data were created or analyzed in this study.
